# Gravity as a Boundary Condition for the Evolution of Three-Dimensional Multicellularity

**DOI:** 10.3390/life16040638

**Published:** 2026-04-10

**Authors:** Oliver Ullrich, Cora S. Thiel

**Affiliations:** 1 Institute of Aerospace Medicine, University of Zurich, Wangenstrasse 68, 8600 Dübendorf, Switzerland; 2 Center for Space and Aviation Switzerland and Liechtenstein, Innovation Park Zurich, Hangar 4, Wangenstrasse 68, 8600 Dübendorf, Switzerland; 3 Space Life Sciences Laboratory (SLSL), Kennedy Space Center, 505 Odyssey Way, Exploration Park, Merritt Island, FL 32953, USA; 4 Institute of Machine Design, Otto-von-Guericke-University Magdeburg, Universitätsplatz 2, 39106 Magdeburg, Germany; 5 Department of Industrial Engineering, Ernst-Abbe-Hochschule (EAH) Jena, Carl-Zeiss-Promenade 2, 07745 Jena, Germany; 6 Faculty of Medical Sciences, Institute of Laboratory Medicine, Private University of the Principality of Liechtenstein (UFL), Dorfstrasse 24, 9495 Triesen, Liechtenstein

**Keywords:** gravity, mechanotransduction, 3D genome organization, chromatin, packing domains, nuclear mechanics, microgravity, hypergravity, multicellularity

## Abstract

Life evolved under a persistent 1 g field that is continuous, ubiquitous, and directionally structured. Here, we synthesize evidence across evolutionary biology, mechanobiology, and genome architecture to propose gravity as a mechanical boundary condition that helped canalize the emergence of complex multicellularity. Order-of-magnitude considerations indicate that gravity-derived hydrostatic loads can fall within force/pressure regimes relevant to nuclear and chromatin mechanosensitivity when transmitted through adhesion–cytoskeleton–LINC–lamina coupling. Comparative genomic and imaging frameworks suggest that complex animals increasingly rely on volumetric genome organization (packing domains and higher-order 3D architectures) that supports durable transcriptional memory and stable differentiated cell identities. Integrating these concepts with altered-gravity experiments, we argue that microgravity and hypergravity perturb chromatin topology and region-level transcription in rapid, largely reversible patterns consistent with a mechanically defined 1 g reference state. We advance a boundary-condition thesis: gravity is not a sole driver but a stable reference that likely contributed to the evolvability and long-term robustness of mechanogenomic architectures required for high-dimensional differentiation and tissue homeostasis.

## 1. Introduction

### 1.1. Gravity as a Fundamental Driver of Evolution

Life on Earth emerged, diversified, and complexified within a gravitational field that has remained essentially constant since the planet first supported biology. Gravity is universally present, continuous, and directionally structured—properties that distinguish it from nearly all other environmental factors considered in evolutionary frameworks. The effective mechanical load experienced by land-dwelling organisms is approximately three orders of magnitude higher than in aquatic environments [1]. It has been emphasized that gravity is among the most stable and continuously present environmental constraints over evolutionary time, thereby acting as a pervasive constraint on biological form and function [2]. Gravity has been described as a persistent evolutionary force that shaped biological structure, physiology, and developmental mechanisms from life’s earliest stages onward [3,4]. This view is consistent with earlier evolutionary arguments proposing that generic physical forces, such as gravity, contributed to the initial organization of primitive multicellular systems and were later progressively assimilated or rendered redundant by genome-encoded regulatory mechanisms [5].

Gravity is seen as a major evolutionary driver in the transition from aquatic to terrestrial life [1], where the move onto land exposed organisms to a nearly thousand-fold increase in mechanical loading compared with water, prompting the evolution of “anti-gravitational” structural solutions such as lignified plant cell walls and mineralized skeletons in animals [1]. This highlights gravity as an obvious long-term shaping force in morphogenesis and tissue architecture. This perspective is supported by classical scale analyses in animal design, which show that gravitational loading imposes size-dependent constraints on support, locomotion, and internal transport systems [2]. Even within aquatic environments, where buoyancy partially counteracts weight, gravitational forces are present and cause hydrostatic pressure, suggesting that gravity influenced biological design from the onset of life on Earth. Historically, gravity has been discussed not only as a physical constant of the terrestrial environment but also as a factor that may have influenced biological form and function over evolutionary time [3,6]. Earlier reviews emphasized organismal adaptation to the persistent 1 g field and the emergence of anti-gravitational structures [6], whereas later work has increasingly reframed gravity in terms of mechanotransduction, cytoskeletal organization, extracellular matrix coupling, and tissue-level force transmission [7]. More recent reviews also note that although the assumption that gravity contributed to biological evolution is longstanding, it remains difficult to test directly because true multigenerational experiments under altered gravity are still limited [3].

At the cellular level, organisms evolved gravity-responsive mechanotransduction pathways and structural systems that influenced morphology, size limits, and energy demands across species [2,5,8]. In particular, early discussions linked gravitational constraints to the emergence of intracellular structural solutions required to maintain spatial order as cellular size and compartmentalization increased [2]. However, evolutionary biology traditionally treats gravity as a static background condition rather than as a physical force that may have actively shaped cellular evolution, genome organization, and the emergence of multicellularity [9,10,11]. In classical neo-Darwinian and molecular evolutionary theory, genetic variation and ecological interaction are emphasized as primary drivers of evolutionary change [11,12], whereas physical constants such as Earth’s gravitational acceleration are rarely invoked as explanatory variables. Because gravity acts continuously on intracellular structures—and, therefore, on the organization of biological matter across domains, from macromolecular complexes and organelles to genome architecture and tissue patterning—the question is to what extent its constant presence contributed to shaping the physical organization and regulatory logic of life. Cells seem to respond strongly to altered gravitational load [13,14,15], whereas organismal outcomes appear more stable [5,16]. This apparent paradox [5,14] has been explicitly discussed in earlier spaceflight studies, which reported robust completion of metazoan development under microgravity despite consistent alterations in cellular proliferation and signal transduction pathways [13,16]. These observations were interpreted as evidence that early gravity-dependent mechanisms had been functionally replaced by autonomous developmental regulatory networks. In this sense, gravity is best understood as an early enabling and constraining factor whose direct developmental role diminished as biological systems evolved autonomous regulatory complexity [5].

### 1.2. Mechanotransduction as an Evolutionary Solution in a Gravity Field

Gravity has been explicitly defined as a vectorial force that assigns weight to biological matter according to mass and orientation within the gravitational field, thereby continuously loading living systems throughout evolution [8], even when organisms inhabit buoyant environments [1,2]. This loading is detectable not only in anatomically large systems but also at the micrometer scale [17,18], where cellular life originated. From this perspective, gravity can be seen as a structural framework that provides persistent spatial information, against which molecular, cellular, and developmental systems evolved. In line with this view, gravity has been proposed as a critical selective pressure driving the evolution of dedicated mechanisms capable of sensing and transducing gravitational forces into biological signals [2,19]. This framing aligns with early proposals that persistent gravitational loading supplied a continuous physical reference that biological structures increasingly internalized through mechanosensitive architectures [2]. Because gravity remained stable across billions of years, evolutionary developments did not need to adapt to fluctuating field strengths but could instead canalize mechanosensitive architectures that rely on consistent downward forcing. This may explain why genome organization, nuclear mechanics, and cytoskeletal function appear so deeply integrated: mechanical responsiveness may have been selected for stability in a constant field. From an evolutionary perspective, gravity has been described as a constant force acting on biology since its origin, shaping biological structure and function across all levels of organization [8].

Supporting this interpretation, developmental systems exposed to microgravity have repeatedly been shown to complete morphogenesis with minimal or no gross defects [16,20], despite clear sensitivity to gravity at the level of isolated cells [15,21,22,23]. This is a paradox explicitly highlighted in spaceflight studies on metazoan development [5,14,24] and interpreted as evidence that early gravity-dependent mechanisms became functionally masked by more robust, genetically encoded developmental programs—an early canalization of mechanically guided processes [5,18].

Consequently, mechanotransduction—the conversion of mechanical signals into biochemical responses—is now widely recognized as a core cellular function of complex organisms: multiple gravity-sensitive biochemical pathways, including cytoskeletal regulators and transcriptional coactivators, have been identified as key mediators of mechanotransductive responses to altered gravitational loading [8,15]. Mechanotransduction has explicitly been framed as an evolutionary adaptation to gravity, enabling cells and tissues to convert persistent mechanical loading into regulatory biological responses [19]. Altered gravity conditions have repeatedly been shown to disrupt mechanotransductive signaling across multiple physiological systems, reinforcing the central role of gravity-dependent force sensing in cellular regulation [3]. Such mechanotransductive capacities may reflect the evolutionary assimilation of processes that were originally influenced by persistent external physicochemical forces—including not only gravity but also matrix stiffness, tensile and compressive stress, shear forces, hydrostatic/osmotic pressure, and spatial confinement—into increasingly autonomous biological regulatory networks [5,25]. Modern cells respond to alterations in gravitational load with changes in cytoskeletal structure [17,26], nuclear shape–lamina coupling [27,28,29], chromatin remodeling and accessibility [30,31,32,33], histone modifications [34,35], and gene transcription [23,36]. Extensive microgravity studies have demonstrated that such gravity-dependent responses affect proliferation, polarity, cytoskeletal organization, signal transduction, and gene expression at the single-cell level [8,19,37]. Comprehensive analyses of immune, musculoskeletal, and cardiovascular cells demonstrate that such gravity-dependent responses are robust, reproducible, and detectable even with short exposure times [3,23,26,34,38,39].

The developmental paradox can be made more informative by focusing on embryogenesis, because embryonic systems provide a uniquely integrated context in which gravity-dependent effects on cellular organization, tissue mechanics, and genome regulation can be assessed simultaneously. Precisely because embryogenesis couples cell division, morphogenesis, lineage allocation, and transcriptional reprogramming within a rapidly changing mechanical environment, it offers an important intermediate level between isolated-cell responses and whole-organism robustness. Embryogenesis constitutes a key biological context in which the influence of gravity on cellular organization, mechanotransduction, and genome regulation can be directly assessed. Experimental literature shows that altered gravitational conditions affect early developmental processes across diverse multicellular systems, including amphibians, fish, and mammals. Spaceflight and ground-based studies indicate that embryonic development can proceed under microgravity; however, this frequently occurs together with deviations in early cleavage dynamics, including altered division symmetry, timing, and blastomere organization, as well as changes in body-axis formation, developmental timing, and programmed cell death. These observations suggest that gravity modulates developmental robustness rather than being absolutely required for embryogenesis [20,40,41,42]. In vertebrate models, particularly amphibians, gravity-sensitive effects have repeatedly been observed during gastrulation and neurulation, indicating that morphogenetic processes dependent on cytoskeletal organization, cell polarity, and coordinated tissue mechanics are especially responsive to changes in mechanical loading [20,41]. In mammalian systems, preimplantation stages appear particularly susceptible, as altered gravity has been associated with reduced blastocyst formation, compromised developmental potential, and shifts in gene expression profiles, pointing to effects on lineage specification and early developmental stability [43,44]. Recent work further supports this view by showing that simulated microgravity can impair fertilization efficiency and early embryonic progression, including developmental competence and lineage allocation [45]. At the mechanistic level, these developmental perturbations have been linked to changes in cytoskeletal architecture, disruptions of mechanotransductive signaling pathways, and downstream modifications in gene expression and epigenetic state, all of which are directly relevant to genome organization and function [14,46,47]. More recent work shows that altered gravity can reshape stem-cell behavior, differentiation trajectories, and transcriptional programs through mechanosensitive and epigenetic mechanisms, thus linking gravitational cues to developmental genome control [48]. Because embryogenesis is fundamentally driven by coordinated changes in gene expression, chromatin architecture, and cell-fate decisions, developmental systems occupy a particularly informative position at the interface of gravity, mechanics, and genome function. Recent reviews have correspondingly emphasized that gravitational biology should be considered in the context of reproduction and embryonic development, where mechanical and genomic regulation are coupled in a highly dynamic manner [49,50]. Taken together, embryogenesis provides a biologically and mechanistically privileged framework for linking gravitational force to multicellular genome regulation.

### 1.3. Physical Forces Acting at the Scale of Single Cells

The emergence of the first cytoskeletal structures can be understood as a response to persistent gravitational forces acting on the earliest complex cells [2]. The gravitational force poses a fundamental organizational challenge for intracellular components: heavier macromolecules, vesicles, and organelles sediment within the cytoplasm, disturbing the stability of the intracellular architecture as the cell volume increases. This effect depends on particle size, viscosity, and timescale (diffusion versus sedimentation), and it becomes increasingly relevant as cellular dimensions and compartmentalization increase. The gravity-driven intracellular stratification, as a core physical problem that would favor the evolution of structural countermeasures, has been discussed explicitly [2]. Under these conditions, the evolution of internal structural frameworks capable of counteracting sedimentation and maintaining spatial organization is favored. Actin filaments and microtubules provided precisely such support, forming the primordial scaffolding that stabilized intracellular positioning and thereby enabled the evolution of larger, compartmentalized eukaryotic cells. In the same conceptual line, gravity-induced sinking has been invoked as an evolutionary pressure favoring active motility systems (e.g., cilia/flagella) to counteract passive sedimentation [2]. Later, as the tensegrity model [18] suggests, this internal framework became mechanically integrated with the extracellular environment through transmembrane adhesion receptors such as integrins. External mechanical loads, including gravitational forces, could now be transmitted via the extracellular matrix to a prestressed cytoskeletal network composed of actin filaments, microtubules, and intermediate filaments. The cytoskeleton has been repeatedly identified as a primary gravity-responsive structure, with altered F-actin organization observed under both real and simulated microgravity conditions [8]. Building on the tensegrity concept, gravity-induced mechanical load has been proposed to modulate mechanotransductive signaling by disturbing the balance between tension and compression at plasma membrane–cytoskeleton interfaces [1]. In this way, gravity not only sets the initial selective conditions for the cytoskeleton’s emergence but also continues to shape its evolution into a mechanosensitive system linking external forces to nuclear and chromatin organization. Mechanosensitive complexes such as integrins, focal adhesions, and ion channels respond within the pN-to-nN range [36,38,39], which corresponds approximately to the static hydrostatic load generated by submillimeter-to-millimeter columns of overlying soft tissue or fluid for a cell of ~20 µm diameter, based on the estimate of ~100 Pa per cm at 1 g [2]. At the upper end, 1–10 nN is sufficient to remodel chromatin at the whole-nucleus scale [27,39,51], corresponding roughly to ~0.3–3 mm of overlying tissue or fluid.

Table 1 organizes experimental force regimes across orders of magnitude, showing that the mechanical landscape relevant to chromatin spans from pN (single-receptor forces) to nN–µN (cell- and tissue-level loads). Single-integrin tension is in the pN range [52], while focal adhesions and whole-cell traction produce nN-scale forces and stresses sufficient to deform nuclei and reorganize chromatin through the cytoskeleton–LINC–lamina continuum [51,53]. Mechanical studies directly demonstrate chromatin mechanosensitivity: force-induced chromatin stretching can rapidly upregulate transcription [30], while nuclear envelope failure modes (e.g., rupture under transient tensile stress) link mechanical loading to genome stability and, potentially, to longer-term epigenomic consequences [54]. Nuclear mechanical response regimes are shaped by both chromatin and lamin A, with distinct chromatin-dominated versus lamin-dominated behaviors [55], consistent with a model in which chromatin is not only a biochemical substrate but also a mechanical element. In tissue contexts, shear stress (Pa range) induces chromatin remodeling and flow-dependent gene regulation in the endothelium [56,57]. Repeated strain can install mechanical memory via chromatin architecture [58], and heterochromatin remodeling can protect the genome from mechanical stress-induced damage [59]. At the high end of physiological loading, chronic compressive stress and elevated interstitial fluid pressure in tumors (kPa range) represent sustained mechanical environments that deform nuclei and alter mechanobiological signaling [60,61,62].

Crucially for gravitational biology, Table 1 highlights that gravity-derived hydrostatic loading is already in the chromatin-relevant range: a fluid or tissue column of ~1 cm yields ~100 Pa pressure differential (≈30 nN on a ~20 µm cell cross-sectional area), establishing a persistent baseline mechanical load [2]. This order of magnitude overlaps with the force regimes that are experimentally sufficient to reshape nuclear architecture and modulate chromatin-linked outputs. The implication is that gravity is not merely a macroscopic “background” but, rather, gravity-derived hydrostatic loading is a background load whose direct impact at the single-cell level is likely subordinate to cytoskeletal and ECM-mediated stresses.

At physiological temperature, thermal energy corresponds to $k_{B}T\approx 4.1$ pN·nm, implying characteristic Brownian force scales of ~4 pN over 1 nm, ~0.4 pN over 10 nm, and ~0.04 pN over 100 nm. By comparison, a gravity-derived hydrostatic load of ~100 Pa, corresponding to a ~1 cm fluid or tissue column, yields ~30 nN on a whole-cell cross-sectional area of ~20 µm diameter, but only ~100 pN over 1 µm^2^, ~10 pN over 0.1 µm^2^, and ~1 pN over 0.01 µm^2^. Thus, gravity-derived loading can overlap with mechanobiologically relevant force ranges at the whole-cell and tissue scales, but it is unlikely to dominate over thermal fluctuations or active intracellular force generation at the smallest subcellular scales.

### 1.4. Gravity and the Emergence of Multicellularity

The transition from unicellular to multicellular life required the coordination of mechanical forces across increasing spatial scales. As organismal complexity increased, gravity increasingly constrained tissue architecture, organ positioning, and fluid distribution, necessitating coordinated mechanotransductive responses across multicellular systems [19]. In multicellular systems, gravity generates positional gradients of hydrostatic pressure and tissue stiffness, requiring cells to decode force cues that vary with location. Comparative perspectives have further suggested that gravity sensing can rely on simple test-mass principles that mechanically load intracellular or membranous structures, providing an evolutionary bridge to dedicated accelerometer organs [2]. Organisms that developed robust mechanosensitive and mechanoregulatory systems could therefore evolve in increasingly complex morphologies. Unicellular eukaryotes such as choanoflagellates and amoebae demonstrate that core mechanosensitive organizational logic emerged prior to the evolution of multicellularity. In these organisms, the need to maintain spatial coherence and intracellular order favored the development of cytoskeletal tensegrity systems and mechanosensitive signaling pathways capable of supporting stable intracellular organization under changing physical conditions. These architectures, comprising actin–microtubule networks, cytoskeleton coupling, and force-responsive regulatory modules, are therefore best understood not solely as mediators of motility or environmental sensing but as general mechanosensitive structural frameworks that support coherent intracellular order. Their specific relevance to gravity likely became more pronounced with increasing body size, tissue stratification, and multicellular force integration. On this foundation, the transition to multicellularity entailed a substantial reconfiguration of cellular and, critically, genomic architecture, including expanded regulatory complexity, stable cell–cell mechanotransduction, and specialized lineage programs. Table 2 summarizes the development of nuclear and chromatin architecture as well as the mechanosensitive 3D regulation potential across unicellular to multicellular organisms. Alongside the evolution of chromatin architecture, mechanosensitive genome regulation modules evolved and became increasingly complex (Table 3 and Table 4).

Bacteria already possess dedicated mechanosensitive ion channels (e.g., MscL/MscS) that respond to membrane tension and osmotic stress [67], showing that mechanosensing is ancient but operates without a nucleus–lamina–chromatin coupling layer. In yeast, canonical nucleosomes and histone modifications exist, and chromosome folding is organized at high resolution (including Micro-C-defined folding principles and TAD-like behaviors) [68,69,70]. However, yeast lacks canonical metazoan lamins and does not exhibit the same lamina-anchored, volumetric 3D genome logic prominent in multicellular animals [71,72]. Thus, unicellular fungi can mount robust mechanostress transcription programs (e.g., via cell-wall integrity signaling) [73,74], yet their genome architecture offers comparatively limited capacity for mechanically controlled long-range repositioning of regulatory DNA at the organismal complexity scale.

Among protists, nuclear and chromatin architectures are highly diverse; many lineages show pronounced 3D genome reorganization linked to developmental transitions or antigenic variation [75,76,77]. Importantly, comparative work argues that 3D genome folding is widespread across the tree of life, but boundary mechanisms differ—with SMC complexes and transcription as deep ancestral “sculptors”, whereas CTCF-based insulation is metazoan-specific [78]. In the lineage closest to animals, choanoflagellates exhibit intron-rich genomes and a metazoan-adjacent regulatory repertoire [79,80], and recent chromatin profiling demonstrates that H3K27 methylation/Polycomb repression is ancestral to animals and their closest unicellular relatives, providing a stable epigenetic repression layer near the animal stem [81]. In parallel, unicellular holozoans such as Capsaspora show enhancer-like regulatory logic [82] and, critically, functional integrin-mediated adhesion, indicating that a key mechanical interface (integrin adhesome–actin cytoskeleton) predated animals [83,84]. A further step toward animal-like mechanoregulation is supported by evidence that Hippo pathway components are required for multicellular morphogenesis in a unicellular relative of animals [85], connecting force-coupled signaling to transcriptional control in a premetazoan context (Table 4).

A decisive architectural transition appears at the base of metazoans: chromatin loops are conserved across early-diverging animals and are absent in unicellular relatives, consistent with the emergence of distal promoter–enhancer control as an ancestral hallmark of animal regulatory genomes [86]. This looping architecture is nested within higher-order genome organization (TADs, compartments, chromosome territories) that becomes increasingly prominent in bilaterians and vertebrates [87,88]. In addition, recent work proposes that noncoding DNA (introns/intergenic segments) encodes volumetric chromatin packing geometry, linking genome sequence composition to stable yet flexible 3D packing domains that scale with organismal complexity [72]. Advanced imaging and packing-domain concepts further support a view of chromatin as a mesoscale polymer system whose geometry can be tuned and reorganized [89,90,91]. Collectively, Table 2, Table 3 and Table 4 support the central idea that mechanosensing is ancient, but the integration of force pathways with stable, high-dimensional genome architecture—including lamina-associated regulation and distal looping—becomes most fully realized in metazoans, enabling mechanosensitive control of gene regulation at the scale required for multicellular differentiation.

**Table 2:** Evolutionary comparison of genome organization, nuclear mechanical coupling, and relevance for gravity-sensitive genome regulation: This table compares broad evolutionary stages from bacteria to bilaterians/vertebrates/humans. For each stage, the second column summarizes the dominant state of genome or chromatin organization, including major features of three-dimensional (3D) genome architecture where present. The third column indicates whether a mechanically relevant nuclear interface is present, including the nuclear envelope (NE), nuclear lamina, lamina-associated organization, and the linker of nucleoskeleton and cytoskeleton (LINC) axis. The fourth column provides the manuscript-specific interpretation of how strongly each stage is expected to support persistent force-coupled control of genome position, chromatin state, and transcription under Earth gravity. Rows are grouped by representative evolutionary stage rather than by individual species, so the entries summarize typical organizational states rather than all exceptions within a lineage. “Premetazoan holozoans” refers here to unicellular animal relatives such as choanoflagellates and Capsaspora. “Volumetric regulation” denotes regulation that depends on large-scale 3D genome packing and nuclear architecture rather than on local promoter-level control alone. LADs, lamina-associated domains; TADs, topologically associating domains; 3D, three-dimensional. The reference column lists representative key sources for each row and is not intended to be exhaustive. Refs, references.

Evolutionary Stage	Genome/Chromatin and 3D Architecture	Nuclear Mechanical Interface	Relevance	Key Refs.
Bacteria (prokaryotes)	No nucleus; DNA is organized as a nucleoid by supercoiling and nucleoid-associated proteins in domains/macrodomain-like structures rather than nucleosome-based chromatin.	No nuclear envelope, lamina, or LINC axis.	Mechanosensing can affect physiology and gene expression, but not through a nucleus–lamina–chromatin mechanotransduction chain.	[92]
Unicellular eukaryotes (yeast and diverse protists)	Nucleosomes and histone marks are present; 3D organization exists (e.g., Rabl-like or lineage-specific domains/compartments), but boundary logic is variable and generally less elaborate than in metazoans.	Nuclear envelope present, but canonical lamins/LAD systems are absent or patchy; NE–chromatin anchoring relies on alternative tethers.	3D chromatin can reorganize and support mechanostress transcription, but stable metazoan-style volumetric regulation remains limited.	[68,69,70,72,75,76,77,80,93,94,95,96]
Premetazoan holozoans (choanoflagellates and Capsaspora)	Animal-adjacent regulatory complexity emerges, including intron-rich genomes, Polycomb repression, and enhancer-like logic; stable distal chromatin loops are still not detected.	Core cytoskeletal and adhesion/mechanotransduction modules are present, while full metazoan lamina/LAD architecture remains incomplete or uncertain.	Regulatory and mechanical modules predate animals, but full loop-based volumetric genome packaging appears later.	[71,79,80,81,82,84,86,96,97]
Early animals	Expanded noncoding/regulatory genome with conserved distal promoter–enhancer and promoter–promoter loops; chromosome-scale topological structure is established.	Canonical metazoan nucleus–cytoskeleton coupling and nuclear envelope architecture are in place.	First clear regime in which mechanics can plausibly reposition and regulate DNA at the whole-genome scale.	[71,86,96,98,99]
Bilaterians/vertebrates/humans	Hierarchical 3D genome with loops, TADs, compartments, territories, and noncoding DNA functioning as a volumetric scaffold.	Mature membrane–cytoskeleton–LINC–lamina–chromatin axis with lamina-associated organization.	Highest mechanosensitive 3D regulatory capacity; gravity and force can rapidly alter nuclear geometry, chromatin topology, and transcription.	[33,36,72,89,90,91,100,101,102,103,104,105,106]

**Table 3:** Evolutionary comparison of force sensing, cell-structural coupling, nuclear relay, and genome-level interpretability across major biological stages: This table summarizes how the capacity to detect, transmit, and interpret mechanical input changes across broad evolutionary stages, from bacteria to metazoans, and relates those changes to the emergence of gravity-relevant genome regulation. The “Force Sensing and Adhesion Context” column identifies the dominant external or cortical structures through which cells interact with physical load, including the bacterial cell envelope, the eukaryotic membrane–cortex interface, substrate- or adhesion-based sensing, and—in metazoans—integrin- and extracellular matrix (ECM)-linked mechanosensing. The “Relay toward the Nucleus” column indicates whether mechanically relevant forces can be propagated through an internal structural network, such as actin, microtubules, intermediate filaments, or functionally analogous structural systems. The “Genome/Nuclear Architecture Context” column summarizes whether mechanical information is expected to reach the genome only indirectly through biochemical signaling, or whether a more continuous path exists that can couple cell mechanics with nuclear shape, chromatin organization, and transcriptional state. Rows are grouped as broad representative stages, not as exhaustive taxonomic inventories, and therefore capture major biological transitions rather than organism-by-organism variation. “Premetazoan holozoans” refers to unicellular relatives of animals, including lineages such as choanoflagellates and Capsaspora, which already show elements of adhesion, signaling, and cytoskeletal complexity relevant to later multicellular mechanoregulation. “Genome relay” is used here broadly to include changes in chromosome packing, chromatin accessibility, nuclear architecture, and transcriptional output. LINC, linker of nucleoskeleton and cytoskeleton; ECM, extracellular matrix. The references listed in the final column are representative sources retained from the original manuscript numbering and support the biological transitions summarized in each condensed row. Refs, references.

Evolutionary Stage	Force Sensing and Adhesion Context	Relay Toward the Nucleus	Genome/Nuclear Architecture Context	Relevance	Key Refs.
Bacteria (prokaryotes)	Membrane-tension channels and envelope or cell-wall mechanics detect physical stress; there is no integrin–ECM system.	Cytoskeletal homologs exist, but there is no actin–LINC–lamina relay and no enclosed nucleus.	DNA is organized as a nucleoid through supercoiling and nucleoid-associated proteins rather than nucleosomal chromatin.	Mechanosensing clearly predates animals, but it does not operate through nuclear mechanocoupling or metazoan-type 3D genome control.	[1,67,107,108,109]
Unicellular eukaryotes (yeast and diverse protists)	Cell-wall-, membrane-, Ca^2+^-, and cytoskeleton-based sensing modules are present; adhesion systems are varied and usually not organized as a stable integrin–ECM network.	A nucleus is present, but force transfer to the genome is indirect and depends on lineage-specific envelope or tethering systems rather than a canonical metazoan lamina axis.	Nucleosomal chromatin and lineage-specific 3D folding are present, but lamina-like systems, LAD-type organization, and metazoan-style boundary logic are incomplete or variable.	This stage supports mechanostress-responsive transcription and chromatin reorganization, yet outside-in volumetric genome regulation remains limited.	[1,3,19,71,73,74,78,83,88,104,110,111,112,113,114,115,116,117,118]
Premetazoan holozoans (choanoflagellates and filastereans such as Capsaspora)	Expanded receptor and adhesion repertoires include cadherin-like and integrin-linked modules, together with specialized cytoskeletal programs that support multicellular-like interactions.	Proto-animal force-signaling pathways can couple adhesion systems with the cytoskeleton, creating a more plausible route from external force to nuclear regulation than in earlier unicellular lineages.	Nuclear architecture is present and regulatory complexity increases, including stable repression programs such as PRC2/H3K27 methylation; higher-order genome folding is ancient even if full metazoan lamina scaling evolved later.	A major pre-animal step appears here: the components needed for force-to-chromatin linkage emerge before true animal tissues.	[3,18,19,71,78,79,81,83,84,85,88,96,104,119,120,121,122,123,124]
Metazoa (early animals to bilaterians/vertebrates)	Mechanosensors such as integrins and piezo operate within cell–cell and cell–matrix adhesions in tissue contexts.	The cytoskeleton, adhesions, LINC complexes, and lamins form a continuous force-transmission pathway from the cell surface to the nucleus.	CTCF-linked boundaries, TADs, LADs, loops, and other higher-order genome features support strong spatial regulation of chromatin and transcription.	This is the first stage with a full membrane-to-chromatin axis capable of persistent gravity-sensitive mechanogenomic regulation across tissues.	[1,3,5,19,30,31,72,83,86,87,88,89,103,104,105,125,126,127,128,129]

**Table 4:** Evolutionary emergence of mechanosensitive hardware and its integration with genome architecture: This table compares major biological stages—bacteria, unicellular eukaryotes, premetazoan holozoans, and metazoa—with respect to the progressive coupling of mechanosensory systems and genome organization. The “Mechanosensitive Hardware” column summarizes the principal structures or pathways available to detect or transmit mechanical input at each stage, including membrane-tension systems, cell-wall or cytoskeletal sensing, integrin–actin coupling, Hippo/Yes-associated protein (YAP)-related signaling, and—in metazoans—the continuous integrin–adhesion–cytoskeleton–LINC–lamina–chromatin axis. The “Genome Architecture State” column indicates whether the genome is organized as a prokaryotic nucleoid or as nucleosomal chromatin with increasingly elaborate higher-order structure. The “Documented or Expected Nuclear/Chromatin Output” column summarizes the main genomic consequences of mechanical input, ranging from altered DNA supercoiling and nucleoid structure to changes in chromatin topology, epigenetic state, and transcription. Because the evidence base is not equally complete across all lineages, this column combines directly documented outputs with phylogenetically grounded expectations where full mechanistic datasets are still limited. Ca^2+^ denotes calcium-dependent signaling; NAPs, nucleoid-associated proteins; LINC, linker of nucleoskeleton and cytoskeleton; LADs, lamina-associated domains; TADs, topologically associating domains. The references listed are representative key sources for each comparative row. Refs, references.

Evolutionary Stage	Mechanosensitive Hardware	Genome Architecture State	Documented or Expected Nuclear/Chromatin Output	Relevance	Key Refs
Bacteria	Membrane-tension channels and envelope/turgor-based responses; cytoskeletal homologs exist, but there is no eukaryotic actin–LINC relay.	Nucleoid organized by supercoiling and NAPs; no nucleus.	Mechanical stress can alter supercoiling, nucleoid structure, and gene expression.	Ancient mechanosensing exists, but without nuclear mechanocoupling or volumetric genome regulation.	[67,130,131,132,133]
Unicellular eukaryotes (yeast and protists)	Cell-wall- or cytoskeleton-based mechanosensing, ancient mechanosensitive channel/Ca^2+^ modules, and variable adhesion systems.	Nucleosomal chromatin and 3D organization are present, but lamina/LAD systems are absent or variable and metazoan boundary logic is incomplete.	Mechanostress transcription and cell-scale mechanosensory programs occur; chromatin effects are mostly indirect or local.	An intermediate stage: 3D chromatin exists, yet stable outside-in control of genome architecture is limited.	[69,70,71,72,73,74,113,115,134,135]
Premetazoan holozoans	Expanded adhesion and cytoskeletal signaling, including integrin–actin coupling and Hippo/YAP-like modules.	Regulatory genome complexity increases (e.g., Polycomb repression and enhancer-like logic), but the full metazoan looping/lamina regime is not yet established.	Epigenetic repression and adhesion-linked mechanosensitive signaling can stabilize cell states.	Immediate pre-animal stage where mechanical hardware and regulatory genome complexity converge.	[79,80,81,82,84,85,136]
Metazoa	Integrin–adhesion–cytoskeleton–LINC–lamina–chromatin axis plus mechanosensitive coactivators.	3D genome with loops, TADs, LADs, and noncoding DNA acting as a volumetric scaffold.	Force can deform the nucleus and chromatin and rapidly alter transcription, chromatin topology, and epigenetic state.	Full mechanogenomic regime compatible with a 1 g-tuned equilibrium and rapid gravity-sensitive genome regulation.	[26,30,31,33,34,35,36,39,72,86,87,103,104,105,129,137,138]

### 1.5. Chromatin Geometry and Metazoan Evolution

A recent landmark publication [72] demonstrated that the linear arrangement of exons, introns, and intergenic DNA encodes the three-dimensional packing geometry of chromatin, providing a physical mechanism whereby genomes generate stable yet flexible transcriptional environments across cell lifetimes. By showing that non-exonic DNA functions as volumetric scaffold that positions exons into an “ideal reaction zone” for efficient transcription, the authors reconcile longstanding paradoxes in epigenetic patterning and propose that the emergence of complex multicellularity was driven not primarily by new genes but by the evolution of genome geometry itself. Taken together, this work reframes introns and intergenic regions as architectural information rather than passive sequence, suggesting a fundamentally structural layer of genomic encoding.

A central challenge in understanding genome function is the question of how a single genome supports hundreds of stable yet modifiable cellular identities across development, aging, and disease, while maintaining efficient transcription over decades in long-lived tissues. It has been demonstrated that nanoscale packing domains (PDs) establish unified chromatin reaction volumes, in which heterochromatin-enriched cores and euchromatin-rich peripheral regions coexist within a continuous density gradient that enables transcription to occur in an intermediate “ideal zone”—a region of intermediate chromatin density within packing domains that is proposed to be most favorable for efficient and stable transcription, and where accessibility and macromolecular crowding are balanced for efficient polymerase activity [89,139,140]. Observations of PDs via ChromSTEM tomography revealed the physical existence of such volumetric packing. It is proposed that the spatial ordering of exons, introns, and intergenic segments encodes the geometry necessary to generate PD reaction volumes, thereby linking linear gene sequence to three-dimensional chromatin architecture. In this framework, exons are positioned so that upon folding of the entire gene body, they fall preferentially within the ideal zone, whereas intronic and intergenic regions contribute the bulk of volumetric DNA needed to build surrounding layers [72,141,142]. Evidence motivating this model includes the longstanding paradox that heterochromatin accumulates within intronic regions of highly expressed, indispensable genes, such as TNNI3 in the human myocardium, despite the requirement for continuous transcription throughout life [143,144]. Rather than being interpreted as transcriptional repression, intron-associated heterochromatin is reinterpreted as a volumetric structural element that is required to sustain PD geometry and maintain exons at ideal reactive positions [72,145].

Thus, non-exonic DNA should scale with exonic content in a manner consistent with volumetric packing geometry, whereas a simple beads-on-a-string organization would produce linear scaling independent of such volumetric constraints. Indeed, an inverse power–law relationship between exon-to-intron ratio and total gene length is observed across human protein-coding genes, consistent with domain-based volumetric packing rather than linear chain behavior [146]. When exon placements are computationally randomized while maintaining the overall sequence content, this power–law relationship collapses toward the null expectation of ~10% exonic content across gene lengths, demonstrating that the empirical pattern is contingent on exon positioning rather than gene length alone [146]. Furthermore, intron length scales as a power law of exon length, with exponents in the range predicted for mass-fractal domains of dimension 2–3, thereby matching the experimentally observed PD geometry [72,90,147].

The same volumetric logic extends beyond single genes to whole chromosomes. Short exon–NE structures, termed “hinges”, have been defined as segments small enough to avoid forming independent PDs but long enough to act as spacing and segmentation elements between domains, thereby enabling the genome to be partitioned into PD-sized volumes [72,90,147]. Hinges are shown to be enriched in CTCF motifs and GC, and they preferentially overlap enhancer marks and active transcriptional features, supporting their proposed role as mechanosensitive and transcription-sensitive boundary elements that are capable of modulating domain division [72,90,147].

ChromSTEM tomography, ChIP-seq, ATAC-seq, nascent RNA sequencing, and polymer interaction data revealed that PD sizes derived analytically from exon–NE segmentation are highly similar to experimentally measured PD volumes, and that polymerase occupancy was enriched on exons and declined across long introns, consistent with the positioning of exons at ideal reaction surfaces and introns in deeper volumetric regions [72,90,139,140,145,147]. Moreover, nascent transcription decreased from 5′ to 3′ within long introns, consistent with the loss of ideal-zone positioning along extended volumetric segments, while hinges displayed greater overlap with active chromatin features and CTCF binding than randomized controls, indicating their likely role in dynamic segment boundary formation. Finally, ATAC accessibility exhibited power–law behavior consistent with the accessibility of outer-domain regions, rather than uniform beads-on-a-string accessibility [72,90,139,140,145,147]. Collectively, these results indicate that transcriptional efficiency and chromatin packing behavior coincide with geometric patterns encoded by exon–NE positioning, even though direct mechanistic perturbation remains beyond current imaging capabilities.

These physical constraints have been shown to have functional consequences for cell identity and disease. Genes defining stable, highly differentiated cell functions tend to be organized in large volumetric assemblies displaying power–law organization, whereas stem-cell regulators and many HOX genes preferentially adopt linear or small-chain geometries, compatible with greater transcriptional plasticity [72]. Notably, the boundary regions between volumetric domains, often associated with hinges, correlate with increased frequency of oncogenic mutations, implying that the structural logic enabling durable gene expression may simultaneously introduce mechanical and replication-associated vulnerabilities, particularly at domain junctions.

### 1.6. Importance of Gravity for the Architecture and Function of Chromatin in Multicellular Organisms

In our own studies, by combining multiple research platforms and cell types, and with validation through inter-mission and inter-platform cross-comparisons, as well as rigorous controls [23,26,36,39,148,149,150], we investigated the role of gravity in shaping spatial chromatin structure and function, specifically targeting those characteristics that define the genomes of multicellular organisms.

We found that, across diverse cell types, short-term exposure to real microgravity consistently triggers rapid and pronounced deviations from the transcriptional and structural equilibrium maintained under Earth’s gravity, revealing that gravitational unloading is sensed and transduced into molecular responses on sub-minute timescales. In myelomonocytic U937 cells, thousands of transcripts changed within seconds, yet nearly all returned to baseline within minutes, indicating an immediate but transient destabilization of transcriptional homeostasis dominated by impaired RNA processing and mRNA turnover [36]. Similarly, primary Jurkat T cells responded within seconds to microgravity phases, where only a focused subset of transcripts were consistently regulated, highlighting a highly selective early transcriptional trigger reflecting chromatin-proximal sensing rather than broad systemic remodeling [148]. Representative early-response transcripts in Jurkat T cells included *ATP6V1A/ATP6V1D*, *IGHD3-3/IGHD3-10*, and the lncRNA *LINC00837*, together with trigger-set transcripts such as *G3BP1*, *KPNB1*, *NUDT3*, *SFT2D2*, and *POMK*, indicating the involvement of vesicular acidification/transport, immune receptor locus regulation, RNA/stress-granule biology, and nuclear transport. These ultra-fast transcriptional changes were reproduced across independent microgravity flight platforms but not under 2D clinorotation, which is consistent with a direct role of altered gravitational loading while also highlighting the limitations of ground-based analogs; thus, the data support the interpretation that altered gravitational loading is a major mechanophysical contributor to chromatin-associated reactions [26,148].

At the chromatin level, microgravity relaxed nuclear architecture and disrupted structural constraints that normally influence transcriptional repression, resulting in rapid transient increases in chromatin accessibility and shifts in intrachromosomal and interchromosomal contact frequencies, particularly within small, gene-rich chromosomes [33]. This localized reshaping of chromatin topology without global compartment switching aligned with reduced anchoring of heterochromatin and diminished H3K9me3-marked promoter repression, mechanically interpreted as loss of lamina-proximal compaction and attenuated transcriptional silencing [150]. Spatial reorganization of chromosomal territories also induced gene positioning toward transcriptionally permissive nuclear interiors, consistent with enhanced intron retention and slower exon usage [150]. Chromatin relaxation was correlated with the rapid activation of gravity-responsive chromosomal regions, emphasizing that transcription without gravity followed spatial remodeling [33,149].

### 1.7. Investigation of Gravitational Conditions Exceeding Earth’s Gravity

In our experiments, hypergravity emerged as a strong, rapidly acting, mechanical perturbation that drives transcriptional, chromatin, and signaling changes before robust adaptation restores homeostasis. At the transcriptome level, U937 myelomonocytic cells showed more than 10,000 altered transcripts after only 20–75 s of hypergravity (1.8–13.5g), far exceeding the breadth of the microgravity response and demonstrating that mechanical overload engages a large early-response program [36]. The overall biological state above Earth’s gravity was dominated initially by upregulation of genes controlling DNA replication, microtubule dynamics, transcriptional regulation, and cell-cycle control, indicating that increased load is interpreted as a proliferative and structural stress signal [36]. Consequently, hypergravity primarily engaged pathways linked to DNA replication, microtubule dynamics, transcriptional regulation and cell-cycle control, followed by marked post-transcriptional remodeling with altered exon usage/intron retention and reduced ribosome biogenesis/RNA-processing output. In Jurkat T cells, launch-phase hypergravity in ballistic rocket missions similarly induced hundreds to thousands of differentially expressed transcript clusters within 75 s, again exceeding the microgravity response and showing that mechanical overloading activates a broader and more intense deviation from the transcriptional equilibrium in Earth’s gravity than unloading [26,148]. Notably, a large fraction of hypergravity-responsive genes were reproducible across suborbital rocket flight and 9g ground centrifugation studies, underscoring that elevated gravitational load is a strong and consistent driver of early transcriptional changes [148].

These early transcriptional deviations are not static but strongly time-structured and reversible, reflecting a characteristic biphasic adaptation pattern reproducibly observed under altered gravitational loading, albeit not necessarily specific to gravity alone. In Jurkat T cells subjected to hypergravity, most transcripts were upregulated after 20 s, but by 75 s this pattern had already begun to invert, and after 15 min the transcriptome was largely normalized or even shifted toward downregulation relative to 1 g, indicating a complete adaptive response back toward or below baseline [39]. This dynamic was mirrored at the post-transcriptional level: after 3 min of 9g, preferentially unspliced transcripts were upregulated, while spliced transcripts were predominantly downregulated, revealing an early surge in transcription not yet processed by the splicing machinery, followed by a rebound phase in which exon usage patterns flipped and many changes reversed by 15 min [149]. Differential exon usage in hypergravity can exceed the number of differentially expressed genes, and protein-coding transcript ratios declined as retained-intron and noncoding isoforms transiently increased, together with strong downregulation of nucleolus-enriched genes involved in ribosome biogenesis and RNA processing [149]. These findings point to a compression-oriented regulatory regime in which rapid transcriptional activation under overload is quickly counterbalanced by post-transcriptional filtering that reduces the coding output and dampens translational capacity, thereby limiting the lasting impact of the initial hypergravity pulse [36,39,149].

At the level of nuclear architecture, hypergravity did not simply mirror the microgravity phenotype but instead established a structurally more compact, radially polarized chromatin state. Nuclear compression under hypergravity strengthened peripheral chromatin tethering and constrained chromosomal mobility, generating a compaction-biased topological configuration characterized by relatively modest increases in intrachromosomal interactions but a reduction in interchromosomal contacts compared with 1 g [33]. In T cells, upregulated genes moved toward the nuclear interior, while downregulated loci shifted toward the periphery, forming a centripetal activation–peripheral repression pattern that was already detectable between 20 s and 3 min and transiently attenuated around 5–7 min before re-emerging, defining a biphasic spatial response with a mechanosensitive transition point [150]. Chromosomal enrichment analyses showed that small, gene-dense chromosomes (16–22) were particularly involved, suggesting that compact territories near the nuclear center were preferentially reorganized to accommodate hypergravity-induced transcriptional programs [150]. In contrast to the pronounced loss of promoter H3K9me3 observed under microgravity, hypergravity largely preserved promoter-proximal H3K9me3, exerting only subtle and stabilizing effects on heterochromatin marks, thereby maintaining lamina-associated repression and chromatin rigidity rather than relaxing it [150]. In this sense, increased gravitational load reinforced chromatin insulation and radial zonation, restraining spatial gene accessibility and buffering the transcriptome against more persistent destabilization.

These findings support a unifying picture in which hypergravity pushes cells into a transiently over-constrained, mechanically compressed state that strongly amplifies early transcriptional and post-transcriptional responses, reinforces chromatin compaction and radial segregation, and acutely modulates signaling modules, followed by rapid adaptation that restores or even overcompensates toward the configuration in Earth’s gravity. Whereas microgravity tends to loosen chromatin, reduce repressive marking, and increase transcriptional permissiveness, hypergravity tightens the nuclear architecture, maintains heterochromatin insulation, and uses extensive exon-level remodeling [23,26,33,36,148,149,150,151,152].

Table 5 frames the observations of nuclear and chromatin dynamics after mechanical or gravitational perturbation in time, showing that mechanosensitive genome regulation operates across multiple temporal layers. Force propagation through prestressed cytoskeletal networks and signaling can occur in sub-seconds to seconds [153], nuclear envelope deformation and viscoelastic relaxation emerge over seconds to minutes [154], and isolated nuclei can actively adapt to force within minutes, demonstrating intrinsic nuclear mechanotransduction [155]. Chromatin stretching can couple with transcription within seconds–minutes [30]. Beyond these rapid effects, chromatin accessibility programs measured by ATAC-seq often manifest over hours to days under sustained mechanical stimulation [156], while the cell cycle and differentiation reconfigure lamina–chromatin relationships over hours to weeks [157,158,159]. This multiscale timing supports a mechanistic division of labor: (i) a rapid mechanical phase that repositions or perturbs chromatin topology, (ii) an intermediate regulatory phase dominated by transcription/splicing and histone modifications, and (iii) long-term stabilization through LAD remodeling and differentiation-linked epigenetic programming.

Chromatin is a physical polymer conformation constrained by nuclear mechanics and embedded in a force-transmitting architecture that begins at the cell surface and ends at the genome. Because external forces reshape cytoskeletal tension and nuclear geometry, they inevitably modulate chromatin’s accessibility, long-range contacts, and transcriptional states [30,31,55,103,104,105,129]. Evolutionarily, mechanosensing predates multicellularity, but metazoans uniquely integrate mechanosensitive “hardware” (integrin adhesions, mechanosensitive channels, force-coupled signaling) with mechanosensitive “software” (distal looping, lamina-associated regulation, volumetric chromatin packing) to achieve stable, reversible, mechanically addressable genome control [72,81,83,86,87,88]. Quantitatively, gravity-derived loading is continuously present in the same range of forces that can remodel nuclei and chromatin [2]. Experimentally, altered gravity induces rapid, reversible transcriptional and 3D genome changes within seconds to minutes [33,36,148].

In the context of gravity, Table 4 and Table 5 emphasize that genome regulation responds at unexpectedly short timescales. Real microgravity and hypergravity exposures in human immune cells induce transcriptome changes within ~20–75 s [36,148], and gravity-dependent 3D chromosomal conformational changes are associated with rapid transcriptional responses in T cells [33]. Hypergravity triggers rapid, transient transcriptome and post-transcriptional adaptations within minutes [34,39], accompanied by coordinated cytoskeletal remodeling and histone-mark changes (e.g., increased H3K9me3) [35].

### 1.8. Earth’s Gravity as a Functional Gravitational Equilibrium

Across these studies, a recurring pattern emerges: Earth’s gravitational force has consistently served as the implicit terrestrial reference framework against which biological organization, regulation, and responses to altered gravity have been interpreted. Building on this view, Earth’s gravity can be understood not only as the environmental baseline of terrestrial life, but also as a biologically active physical constraint that stabilizes cellular organization. It has been emphasized [160] that gravity sensing evolved repeatedly across diverse lineages, indicating that gravity has long acted as a pervasive selective and organizational factor. At the cellular level, graviperception is consistently coupled to mechanical asymmetry, for example, through statolith-like structures, denser intracellular compartments, or the whole cell body, which transmit force to structures such as the endoplasmic reticulum and cytoskeleton and thereby initiate downstream signaling [160]. In parallel, Bizzarri et al. [161] propose that gravity acts as a canalizing constraint on cell states: under microgravity, cells can access a broader range of phenotypic configurations, whereas return to 1 g promotes re-stabilization of a preferred organizational state. In this formulation, gravity does not directly instruct cellular identity, but it shapes the physical context in which regulatory states are stabilized. This provides an important transition to the genome, because it suggests that genomic activity is deployed within a mechanically constrained cellular architecture in which gravity helps delimit, bias, and stabilize the range of possible transcriptional and phenotypic outcomes [161].

Earth’s gravity appears to function as a stable terrestrial reference condition for transcriptional regulation, spatial chromatin organization, and mechanosensitive signaling, whereas deviations toward microgravity or hypergravity induce rapid and often reversible perturbations [33,36,148]. Long-term exposure to microgravity consistently reveals physiological deconditioning, supporting the interpretation of 1 g as the gravitational reference state under which terrestrial life optimized its structural and regulatory systems [3,19]: At the transcriptional level, 1 g maintains homeostasis and balanced gene-regulatory output, while both microgravity and hypergravity immediately and reversibly disrupt this equilibrium [26,33,36,148,149,150]: 1 g preserves heterochromatin boundaries, radial genome compartmentalization, and stable promoter repression. Whereas microgravity selectively weakens H3K9me3-dependent silencing and increases chromatin accessibility, shifting loci toward transcription-favoring spatial configurations [150], hypergravity stabilizes or minimally perturbs these repressive states, reinforcing spatial segregation and directing activated genes inward and repressed loci outward, in line with mechanical compression of nuclear architecture [33,150]. These opposing spatial signatures are consistent with the view that chromatin topology in terrestrial cells is organized relative to the 1 g condition, although the current data do not establish gravity as the sole or specific determinant of these responses [26,33,36,150]. Thus, 1 g seems to be a mechanically defined anchor point. Even when gravitational deviations induce dramatic transcriptional or signaling changes [23,151,152], the system’s intrinsic buffering capacity frequently restores gene-regulatory and chromatin-associated states toward the 1 g baseline within minutes, supporting the interpretation that many terrestrial cell systems are functionally adapted to operate around that condition. This is consistent with the idea that cellular architecture is adapted to, and rapidly recovers toward, the 1 g condition [33,36].

Gravity is increasingly conceptualized not as a direct regulator of genome activity, but as a persistent physical boundary condition that acts on cellular architecture and is subsequently propagated into genome function through mechanotransductive coupling. Across the current literature, a hierarchical model has been delineated in which altered gravity is proposed to perturb cytoskeletal self-organization, cellular geometry, and intracellular force balance at an early stage. These structural perturbations are then transmitted through nucleo-cytoskeletal linkages to the nucleus, where changes in nuclear mechanics, chromatin compaction, and higher-order genome organization are induced, with downstream consequences for transcriptional and epigenetic regulation: A foundational physical basis for this view was provided by the demonstration that microgravity disrupts higher-order microtubule self-organization, indicating that gravity can influence the structural substrate upon which cellular order is established [162]. This interpretation has been extended by mechanobiological frameworks in which external physical forces are transmitted through the cytoskeleton and nuclear interfaces to reshape chromatin organization, epigenetic state, and gene expression, while reciprocal feedback from chromatin architecture to nuclear and cellular mechanics has likewise been emphasized [31,163]. In parallel, it has been argued that the folded genome should not be regarded as a passive target of mechanical input, but rather as an active determinant of nuclear rigidity, spatial organization, and regulatory behavior, such that genome architecture itself participates in the interpretation of mechanical environments [33,164]. Within this conceptual framework, it has been shown that gravitational change is associated with broad transcriptional responses in mammalian and plant systems, including pathways related to mechanotransduction, metabolism, proliferation, immunity, growth, and cellular architecture [165,166], and it has further been discussed that these responses extend beyond transcript abundance alone to encompass chromatin-associated and epigenetic alterations [33,34,35,167]. Particularly strong support for a structural–functional coupling model was provided by the demonstration that simulated microgravity was associated with altered higher-order genome organization, chromosome packing and differential gene expression in Klebsiella [168], and that gravitational force-induced 3D chromosomal conformational changes are associated with rapid transcriptional response in human T cells [33], thereby directly linking genome architecture to genome function under altered gravity. This view has been reinforced by broader organismal and translational analyses in which gravity-sensitive effects on genome function were described across multiple physiological systems in Drosophila [169]. Further studies are currently underway to resolve this relationship more directly, including investigations of hypergravity-induced changes in chromatin conformation and nuclear structure in cardiomyocytes and the establishment of a new experimental platform based on random positioning machine to examine, in living human cells, how simulated microgravity affects genome organization and chromatin dynamics [170,171].Taken together, these findings support the interpretation that gravity modulate the physical organization of the cell, and this altered mechanical state is then converted into changes in chromatin architecture, nuclear mechanics, and ultimately transcriptional and epigenetic output. On this basis, Earth’s gravity may be regarded as a long-standing mechanical reference condition under which cytoskeletal organization, nuclear architecture, and genome function have been co-stabilized over evolutionary time, ensuring mechanosensitive resilience across physiological contexts [23,150,151].

### 1.9. Current Knowledge and Unresolved Questions

Taken together, existing studies demonstrate that gravity influences cytoskeletal organization, mechanotransduction, and gene expression across biological systems, including during embryogenesis. These findings establish that gravity-dependent effects on cellular and developmental processes are robust and reproducible across multiple model systems. However, these observations are typically interpreted within specific experimental or biological contexts and remain fragmented at the level of individual pathways, cell types, or developmental stages. A general conceptual framework that integrates these findings and explains how persistent gravitational loading relates to higher-order genome organization and the emergence of multicellular complexity is still lacking, in particular how gravity-dependent mechanical constraints may have contributed to the stabilization and long-term robustness of three-dimensional genome architecture in multicellular organisms. In the following, we therefore propose a hypothesis that integrates gravity in the mechanogenomic framework.

### 1.10. Gravity as a Boundary Condition for the Evolution and Stabilization of Mechanogenomic Architecture

Below, a theoretical integrative framework is proposed in which gravity is treated not as a unique cause of chromatin organization, but as a persistent physical boundary condition that may have facilitated the evolution, stabilization, and reproducible function of force-coupled three-dimensional genome architecture in multicellular organisms. This framework extends current assumptions by linking three lines of evidence that have largely been discussed separately: first, the broad evolutionary increase in volumetric genome organization with increasing organismal complexity [72,172]; second, the known mechanical addressability of chromatin through the integrin–cytoskeleton–LINC–lamina–chromatin continuum; and third, the observation that altered gravitational loading can rapidly perturb chromatin-associated reactions, nuclear architecture, and transcriptional regulation [23,26,33,36,148,149,150,151,152]. The central hypothesis is therefore functional rather than ontological: gravity is not proposed to generate chromatin architecture de novo, but to have contributed to the long-term stabilization of a mechanical operating regime within which metazoan three-dimensional genome organization could become robustly coupled to durable differentiated gene expression.

This hypothesis starts from a simple evolutionary premise. Gravity acted continuously throughout biological evolution as a directional physical constraint that shaped form, support systems, body axes, and mechanotransductive organization. Its relevance became particularly pronounced during the transition from aquatic to terrestrial life, when increased mechanical loading favored the evolution of load-bearing structures such as plant cell walls and animal skeletons. Yet classical evolutionary theory has rarely considered gravity as an active explanatory variable at the level of intracellular organization. Here, it is proposed that this omission may be important, because the emergence of complex multicellularity required not only morphological support and tissue mechanics, but also stable nuclear and genomic architectures capable of preserving lineage-specific transcriptional states over long timescales. In this view, gravity becomes relevant, because it imposes a continuous, directional, spatially cumulative mechanical background that becomes increasingly consequential in larger, fluid-coupled, mechanically integrated multicellular systems [2].

The key conceptual step is to distinguish between the existence of three-dimensional chromatin organization and its functional integration into multicellular regulatory logic. Three-dimensional genome folding, domain organization, and long-range chromatin interactions are ancient and broadly distributed features of eukaryotic genome organization. What appears to have changed during evolution is not the existence of 3D chromatin architecture itself, but the extent to which it became coupled to lamina-associated anchoring, radial chromosome organization, distal enhancer–promoter communication, volumetric packing-domain geometry, and large noncoding structural scaffolds within differentiated multicellular tissues. In metazoans, therefore, genome architecture may be understood as a further elaboration and stabilization of older eukaryotic chromatin-organizational principles under the conditions of multicellularity. Particularly important in this context is the observation that the evolutionary transformation of genome architecture parallels increasing organismal complexity: *S. cerevisiae* predominantly exhibits a linear beads-on-a-string organization, *C. elegans* shows a mixture of linear and volumetric architectures, whereas *D. melanogaster*, *D. rerio*, *M. musculus*, and humans overwhelmingly display power–law volumetric genome organization at both gene and chromosomal scales [72]. This progression suggests that the decisive genomic innovation associated with complex multicellularity may not have been the acquisition of entirely new genes, but the transformation of genome geometry itself into a volumetrically encoded regulatory substrate capable of stable transcriptional memory, partitioned reaction spaces, and durable cell-type specialization [72,172].

From this follows the central mechanogenomic proposition of the present framework: in metazoans, stable genome function increasingly depends on a mechanically maintained spatial operating regime. In such a regime, lamina-associated chromatin tethering, radial chromosome positioning, chromatin compaction, and packing-domain geometry collectively preserve reproducible transcriptional states. Because these features are mechanically addressable through the cytoskeleton–LINC–lamina–chromatin relay, and because Earth’s constant 1 g field contributes a continuous tissue-scale and hydrostatic background load, long-term evolution under 1 g may have favored mechanogenomic architectures that remain robust under continuous gravitational loading. On this view, gravity is best understood as a reference condition for the stable operation of force-coupled genome architecture in multicellular tissues. Its biological significance would then lie less in providing a singular intracellular force signature than in helping to define the persistent mechanical baseline within which chromatin architecture functions reproducibly. This is why the expected relevance of gravity should increase not at the scale of isolated molecules or isolated single cells, but at the transition to multicellularity, where tissue height, hydrostatic gradients, long-range force transmission, and mechanically coupled body axes emerge [2,3,4,5].

This also clarifies why altered-gravity findings are mechanistically informative. If chromatin architecture is part of a mechanically integrated regulatory system, then deviations from 1 g should perturb not only general cell physiology, but preferentially those nuclear features that are central to metazoan genome function: lamina-proximal repression, chromatin compaction, radial genome order, long-range contact topology, and the transcriptional balance associated with volumetric chromatin packing. The altered-gravity literature is consistent with exactly this expectation. It has been shown that chromatin-associated reactions respond rapidly to changes in gravitational loading [26,148] and that transcriptional and three-dimensional genome changes can arise within seconds to minutes [33,36,148]. These observations argue against a purely generic stress interpretation. Their very rapid onset, partial reversibility, and preferential involvement of nuclear and chromatin-linked features are more consistent with a model in which altered gravity perturbs force transmission into the nucleus and thereby shifts chromatin away from a mechanically maintained transcriptional operating regime [23,26,33,36,148,149,150,151,152]. In this interpretation, the gravity-free state is a mechanically deregulated condition in which chromatin organization, transcription, splicing, adhesion, and surface signaling become perturbed in coordinated, cell-type-specific patterns [23,26,33,36,148,149,150,151,152].

The present framework therefore organizes the argument on three explicitly distinct levels. First, at the observational level, microgravity and hypergravity are associated with rapid and partly reversible changes in chromatin organization, nuclear architecture, and transcriptional output [33,36,148,152]. Second, at the mechanistic level, these effects are interpreted as consequences of altered force transmission through the integrin–cytoskeleton–LINC–lamina–chromatin axis, with resulting perturbation of lamina coupling, radial genome organization, chromatin compaction, and higher-order contact topology. Third, at the causal-predictive level, a decisive experimental expectation follows: if this mechanogenomic model is correct, then targeted disruption of the force-transmitting relay should attenuate, delay, or abolish gravity-sensitive chromatin and transcriptional responses. The model therefore predicts not catastrophic loss of genome function outside 1 g, but rapid displacement from a homeostatic chromatin operating regime, followed by partial and context-dependent compensation [33,36,148,152].

This logic also sharpens the meaning of 1 g as a biological reference state. The equilibrium referred to here is not thermodynamic equilibrium, but a functional mechanobiological reference condition in which cytoskeletal tension, lamina-coupled chromatin organization, chromosome territory positioning, and transcriptional output remain sufficiently stable to support reproducible cellular homeostasis. Under normal Earth gravity, continuous loading contributes to the tensile balance that constrains chromatin compaction, architectural compartmentalization, and inter-territorial distances. This favors stable chromosome territory positioning, in which gene-rich chromosomes occupy more central, transcriptionally permissive nuclear environments, whereas gene-poor chromosomes and lamina-proximal regions remain associated with repressive nuclear zones. The observation that chromosomes 18 and 19 systematically shift their expression states depending on the gravitational environment is consistent with this principle and with their differential radial positioning [33]. Under this model, unloading would be expected to relax chromatin and weaken structural constraint, whereas overloading would be expected to reinforce compaction and peripheral tethering, with both directions of deviation displacing the genome from its optimal spatial operating range [33,36,148,152].

The resulting hypothesis proposes that the evolution of complex multicellularity involved the progressive coupling of genome regulation to mechanically stabilized three-dimensional chromatin architectures, and that this coupling emerged under the persistent boundary condition of Earth’s gravity. Gravity would thus not be required for chromatin to exist, nor would it be sufficient on its own to determine genome organization. Rather, it may have contributed to the evolutionary stabilization of the narrow spatial and mechanical operating range within which force-coupled genome architecture can support durable lineage-specific transcription. In this sense, Earth’s gravity may be regarded as a long-standing directional reference condition that helped co-stabilize tissue mechanics, nuclear architecture, and volumetric genome organization over evolutionary time [72,172].

If correct, this model yields clear and testable predictions. Altered gravity should rapidly perturb chromatin topology and transcription. These responses should depend on an intact cytoskeleton–LINC–lamina–chromatin relay, and their magnitude should scale with the degree to which this mechanically integrated relay has been elaborated across lineages. More broadly, the model predicts that the strongest gravity-sensitive effects should not occur in those genomic features that are least structured, but in those most central to metazoan chromatin function: lamina-associated repression, packing-domain geometry, radial chromosome order, and the long-range contact architecture that supports durable differentiated transcription. The altered-gravity data available so far do not constitute definitive proof of evolutionary causality. They do, however, support the biological plausibility and experimental accessibility of the proposed force-to-chromatin pathway [26,148,152]. The working hypothesis advanced here is therefore that Earth’s 1 g field helped stabilize the mechanogenomic regime in which volumetric chromatin organization could become an evolvable and robust substrate for complex multicellularity.

### 1.11. Gravity and the Stabilization of Geometrically Encoded Chromatin

If Earth’s gravity is considered a long-standing mechanical reference condition for force-coupled genome function, a further question follows: under which structural principles might such a reference state have become biologically stabilized over evolutionary time? Within the framework of geometrically encoded chromatin organization, gravity may be more precisely regarded as a persistent physical boundary condition within which genome geometry not only emerged, but became functionally stabilized. Such a view is consistent with the broader proposition that gravity continuously shapes biological structure across scales, thereby linking genome function, tissue mechanics, and organismal physiology within a common physical framework [3], as well as with earlier scale-based arguments that gravitational loading constrains feasible biological architectures from intracellular ordering to the whole animal [2]. In this perspective, Earth’s 1 g field is not interpreted as a direct determinant of chromatin forming, but as part of the enduring mechanical background against which nuclear organization operates reproducibly. At 1 g, continuous force transmission through the cytoskeleton–LINC–lamina–chromatin axis may help maintain the nucleus within a defined mechanical regime that constrains chromatin compaction, radial chromosome positioning, and inter-territorial spacing. The rapid and partly reversible shifts in chromosome territories, chromatin contacts, and coordinated transcriptional responses of gravity-responsive chromosomal regions (GRCRs) observed under both reduced and increased gravitational loading are therefore consistent with the interpretation that the terrestrial 1 g state constitutes a long-term functional reference condition for chromatin organization [33].

The geometrically encoded chromatin model adds a second layer to this argument by suggesting that the genome is internally organized according to geometric rules that generate nanoscale packing domains (PDs) characterized by a heterochromatic core, an optimal transcriptional zone, and an outer euchromatic shell [72]. Within this model, non-exonic DNA serves as volumetric scaffolding, whereas exons are projected into density regimes most favorable for productive transcription. The finding that most human genes obey a power–law relationship between exon and intron length, and that chromosomes can be decomposed into power–law segments separated by hinge elements enriched for CTCF motifs, suggests that packing-domain geometry is itself encoded in genome structure and contributes to the generation of stable reaction volumes capable of sustaining transcriptional memory over long timescales [72]. The question that follows is, therefore, under which physical conditions this order can be assembled, preserved, and functionally exploited with sufficient reproducibility. In this context, gravitational loading may be considered one component of the broader mechanical environment that stabilizes chromosome-territory organization at larger scales and packing-domain geometry at smaller scales [72].

Complex multicellularity did not arise in a mechanically neutral world, but under the continuous directional constraint of Earth’s gravity. As metazoan body structures, tissue specialization, and physiological integration increased, durable cell-type-specific transcriptional states had to be generated repeatedly from an invariant genome. Geometrically encoded chromatin packing domains provide a plausible solution to this problem, because they enable a single linear genome to be folded into multiple stable regulatory states [72]. Yet the reproducible self-assembly and long-term functional stability of such volumetric domains are unlikely to be entirely independent of mechanical context. Rather, they would be expected to depend on a sufficiently stable regime in which fractal packing, density gradients, hinge-mediated segmentation, and radial nuclear order remain robust over time. Earth’s constant 1 g field may have provided such a regime. On this view, selectable variation would not have resided in gravity itself, but in biological architectures that differed in their capacity to preserve spatial and regulatory stability under that persistent condition [72]. Geometrically encoded chromatin may thus be understood as an internal organizational logic for transcriptional memory, whereas 1 g may have supplied a stable external physical reference condition within which this logic became robust, scalable, and evolvable.

This interpretation further places intracellular structural evolution into a broader mechanobiological continuum. Under constant gravitational loading, the maintenance of precise spatial relationships among organelles, macromolecular assemblies, and reaction zones would have favored the emergence of intracellular scaffolds, tension-bearing cytoskeletal systems, and chromatin–lamina linkages capable of preserving molecular organization in three-dimensional space. These architectures, initially relevant for maintaining intracellular order under load, may subsequently have provided the structural substrate for multicellular mechanosensitivity and tissue-level gravity responsiveness. Within the nucleus, the same principle can be extended to geometrically encoded chromatin domains: loop trajectories, enhancer–promoter proximity, lamina association, and compaction gradients all depend on sufficiently stable three-dimensional embedding. Cytoskeletal and lamina-linked tension systems may therefore function as buffers and relays that help preserve radial genome order and chromatin packing during changes in posture, movement, or altered environmental loading.

Taken together, these considerations suggest that gravity-linked tension may form part of the mechanically maintained reference regime that preserves transcriptional identity in multicellular systems. This proposition remains hypothetical, but it is experimentally tractable and logically continuous with the mechanogenomic framework developed above. A central prediction follows directly: if gravity-sensitive genome function is mediated through the cytoskeleton–LINC–lamina–chromatin continuum, then disruption of this force-transmitting relay during altered-gravity exposure should attenuate the stability, reproducibility, or recovery of cell-type-specific transcriptional programs. In this formulation, gravity is not proposed as a unique cause of genome geometry, but as a persistent physical condition that may have contributed to stabilizing the spatial and mechanical regime within which geometrically encoded chromatin became capable of supporting durable multicellular gene regulation [72].

## 2. Conclusions

Taken together, the evidence synthesized here supports a specific mechanogenomic causal hypothesis. We propose that, in metazoans, stable three-dimensional genome function operates within a mechanically defined 1 g conditioned reference state. The constant 1 g field may have formed part of the developmental and evolutionary physical context in which spatial genome encoding became sufficiently stable, interpretable, and scalable to support differentiation, lineage fidelity, and tissue organization. Conversely, gravity-responsive elements evolved to stabilize precisely those chromatin architectures that encode regulatory identity, ensuring that mechanical perturbations do not erase transcriptional memory. Thus, multicellularity rests upon gravity’s dual role as both constraint and stabilizer: gravitational load created the need for high-order intracellular geometry, and graviresponsive systems maintain that geometry as organisms become more complex. Life did not adapt to gravity after achieving complexity - selection under persistent 1 g may have favored genome architectures that supported robust multicellular complexity. Thus, gravity can be viewed as an important enabling principle of multicellular life on Earth, a potential biophysical enabling factor that may have helped render the evolution, stabilization, and persistence of complex life possible in the first place. In this sense, gravity has been argued to shape not only the space–time continuum but also a biological continuum, integrating physical forces with evolutionary and physiological processes. Gravity may have constituted a persistent physical condition that may have favored and may continue to stabilize the functional operating range of complex metazoan mechanogenomic architectures, which, in turn, could have been an important prerequisite for the development of multicellular life.

## Figures and Tables

**Table 1:** Quantitative mechanical force regimes relevant to nuclear and chromatin mechanosensitivity: The table compares representative extracellular, cellular, and nucleus-directed mechanical inputs by cue/context, typical magnitude, duration, biological setting, and reported nuclear or chromatin-linked response. Force and stress units are reported as pascal (Pa), kilopascal (kPa), nanonewton (nN), piconewton (pN), dyne per square centimeter (dyn/cm^2^), millimeter of mercury (mmHg), micrometer (µm), millisecond (ms), second (s), hour (h), and hertz (Hz); values are literature-derived approximate ranges intended as order-of-magnitude comparisons rather than directly normalized measurements across one assay platform. The cell or tissue column identifies the experimental context in which the response was described, and the response column summarizes reported effects on nuclear deformation, chromatin remodeling, transcription, or genome-protective adaptation. Gravity-derived hydrostatic loading is included as the estimated continuous baseline at 1 g for a fluid or tissue column. IFP, interstitial fluid pressure; AFM, atomic force microscopy; MSC, mesenchymal stem cell; LINC, linker of nucleoskeleton and cytoskeleton. Refs, references.

Mechanical Cue/Context	Typical Magnitude (Range)	Typical Duration/Waveform	Cell or Tissue Type	Observed Nuclear/Chromatin-Linked Responses	Refs.
Gravity-derived hydrostatic loading (fluid/tissue column)	Estimate: ~100 Pa per cm fluid at 1 g (≈30 nN on a ~20 µm cell cross-section per cm)	Continuous (static baseline); scales with posture, tissue height	Any cell embedded in fluid/tissue; stronger gradients in thicker tissues	Constant baseline load; can add to cell-generated forces and influence long-term tissue/nuclear shaping	[2]
Single-molecule integrin tension (ligand-bound integrins)	pN scale per integrin (~1–10 pN)	ms–s; fluctuating	Adhesion sites in adherent cells	Molecular force thresholds gate downstream mechanotransduction (upstream of LINC/lamina/chromatin)	[52]
Focal adhesion/local adhesion traction	Local stresses can reach ~kPa scale; summed forces per adhesion often nN scale	Seconds–minutes; dynamic remodeling	Fibroblasts, epithelial cells, MSCs, many adherent cell types	Cytoskeleton–LINC–nucleus force transfer; promotes nuclear deformation and chromatin remodeling	[51,53,63]
Whole-cell traction (traction force microscopy)	Traction stresses commonly ~10–10^3^ Pa (context-dependent); net forces often 10–10^3^ nN	Minutes–hours; sustained or fluctuating	Migrating/spreading adherent cells	Traction level correlates with nuclear shape change and force transmission to chromatin via LINC/lamina	[51,53]
Direct chromatin stretching (probe-based)	nN-scale perturbations reported to directly modulate transcription (~0.1–10 nN depending on geometry)	Sub-second to seconds; pulses	Cultured mammalian cells (experimental manipulation)	Direct chromatin stretching can rapidly increase transcription; indicates “mechanical access” to genome regulation	[30,53]
Local tensile stress at nuclear envelope (direct nuclear probe)	Brief local stress leading to ~1% strain; transient application (~0.2 s)	~0.2 s pulses	Mammary epithelial cells (example in study)	Transient tensile deformation can rupture the nuclear envelope; impacts genome integrity/regulation	[54]
Micropipette aspiration/nuclear mechanics	Effective pressures typically kPa range (assay-dependent)	Seconds–minutes	Isolated nuclei or intact cells	Quantification of lamina/chromatin contributions to nuclear deformability; connects mechanical load to nuclear rearrangements and mechanosensitive signaling	[64,65]
Nuclear micromanipulation	Often ~1–10 nN (nuclear deformation)	Seconds–minutes	Mammalian cells/isolated nuclei	Chromatin- vs. lamin-dominated nuclear mechanics	[55,64]
AFM indentation/compression (cell or nucleus)	Forces commonly ~0.1–100 nN (setup- and cell-dependent)	Seconds; repeated probing	Many cultured cell types	Controlled indentation links applied force to nuclear strain and chromatin state changes	[66]
Shear stress (blood flow/perfusion)	Physiological endothelium often ~5–25 dyn/cm^2^ (=0.5–2.5 Pa)	Minutes–hours; steady or pulsatile	Endothelial cells	Shear flow can remodel chromatin	[56,57]
Cyclic stretch/strain (tissues, engineered stretch devices)	Often ~5–20% strain, ~0.1–1 Hz (platform-dependent)	Minutes–days	Fibroblasts, MSCs, muscle/connective tissue models	Chromatin architecture can encode mechanical memory; heterochromatin remodeling helps protect the genome	[58,59]
Solid compressive stress in tissues/tumors	Reported growth-inhibiting stress ~45–120 mmHg (=~6–16 kPa)	Hours–days (chronic)	Multicellular spheroids/tumors	Chronic compression sustains nuclear deformation and can alter growth and tissue architecture	[60,62]
Interstitial fluid pressure (IFP), especially tumors	Often elevated, e.g., ~10–40 mmHg (=~1.3–5.3 kPa)	Hours–days (chronic)	Solid tumors; pathological tissues	Sustained pressure acts as a tissue-level load influencing mechanotransduction and transport	[61,62]

Note: A ~20 µm diameter cell (area ~3.1 × 10^−10^ m^2^) experiences ~30 nN from ~100 Pa.

**Table 5:** Temporal framework for nuclear and chromatin responses to altered mechanical or gravitational loading: This table organizes mechanogenomic responses by approximate time window, from sub-second events to longer-term remodeling over hours to weeks, to show how gravity-relevant responses can emerge across multiple biological timescales. The “Time Window” column provides broad response bins rather than universal cutoffs, because the underlying studies differ in organism, cell type, exposure system, loading profile, and readout. The “Dominant Response Layer” column indicates the principal level at which regulation is expected to dominate within each interval, beginning with immediate signal propagation, followed by intrinsic nuclear mechanotransduction, then adaptive regulatory responses, and finally durable genome remodeling. The “Representative Nuclear/Chromatin Readouts” column lists typical outputs reported or inferred for that time range, including nuclear deformation, chromatin stretching-linked transcription, chromosome-territory or 3D genome shifts, exon-usage or splicing changes, histone-mark changes, chromatin accessibility programs, and lamina-associated domain (LAD) resetting. The “Typical Adaptation or Reversibility” column indicates whether the response is generally transient, biphasic, rapidly normalizing, or capable of stabilizing into longer-lasting memory or reprogramming. The “Gravity-Relevant Examples” column highlights representative altered-gravity observations that illustrate how the proposed gravity-sensitive reference state may be perturbed over time; these examples are comparative illustrations and do not imply statistical pooling or cross-study normalization. “3D genome” refers to higher-order spatial genome organization within the nucleus, and approximate expressions such as “about 1 h” are intended as biologically useful bins rather than exact thresholds. The references listed in the final column identify representative key studies for each temporal class. Refs, references.

Time Window	Dominant Response Layer	Representative Nuclear/Chromatin Readouts	Typical Adaptation or Reversibility	Gravity-Relevant Examples	Key Refs
Sub-second to seconds	Immediate mechanical propagation	Prestressed cytoskeletal signal transmission and the first nuclear envelope/nuclear deformation events.	Very fast; often acts as the trigger for later transcriptional and chromatin programs.	Real microgravity/hypergravity studies detect the earliest transcriptome “trigger sets” within about 20–75 s, consistent with perturbation of an already preloaded system.	[36,148,153,154]
Seconds to minutes	Intrinsic nuclear mechanotransduction and direct chromatin response	Nuclear stiffening/adaptation, chromatin stretching-linked transcription, and early 3D genome or chromosome-territory shifts.	Often transient or biphasic; recovery depends on exposure length.	Rapid altered-gravity responses include early transcriptional changes and 3D chromosomal conformational changes in immune cells and T cells.	[30,33,155]
Minutes to about 1 h	Adaptive regulatory phase	Splicing/exon-usage shifts, histone-mark changes, cytoskeletal recovery, and transcriptome inversion/normalization.	Many changes attenuate within minutes; some cytoskeletal features recover in the order of ~1 h.	Hypergravity induces exon-usage changes by ~3 min, histone/cytoskeletal co-responses within minutes, and substantial normalization by ~15 min.	[34,35]
Hours to weeks	Stabilization and long-term genome remodeling	Chromatin accessibility programs, cell-cycle rebuilding of 3D genome architecture, LAD resetting, and differentiation-linked lamina reorganization.	Supports durable stabilization, memory, or reprogramming under sustained loading histories.	Defines the longer remodeling window expected after persistent altered mechanical or gravitational conditions.	[156,157,158,159]

## Data Availability

No new data were created or analyzed in this study. Data sharing is not applicable to this article.

## Abbreviations

The following abbreviations are used in this manuscript:

1 g	Earth gravity (normal gravitational reference level)
2D	Two-dimensional
3D	Three-dimensional
AFM	Atomic force microscopy
ATAC	Assay for Transposase-Accessible Chromatin
ATAC-seq	ATAC with sequencing
Ca^2+^	Calcium ion
ChIP-seq	Chromatin immunoprecipitation with sequencing
cm	Centimeter
CTCF	CCCTC-binding factor
DNA	Deoxyribonucleic acid
ECM	Extracellular matrix
F-actin	Filamentous actin
FtsZ	Bacterial cell-division protein (tubulin homolog)
GC	Guanine–cytosine (GC content)
GRCRs	Gravity-responsive chromosomal regions
H3K27me3	Histone H3 lysine-27 trimethylation
H3K4me3	Histone H3 lysine-4 trimethylation
H3K9me3	Histone H3 lysine-9 trimethylation
Hi-C	Chromosome conformation capture (Hi-C method)
HOX	Homeobox gene cluster/family
Hz	Hertz
IFP	Interstitial fluid pressure
kPa	Kilopascal
LAD(s)	Lamina-associated domain(s)
LINC	Linker of nucleoskeleton and cytoskeleton (complex)
MAPK	Mitogen-activated protein kinase
Micro-C	Micrococcal nuclease-based chromosome conformation capture
mm	Millimeter
mmHg	Millimeters of mercury
MreB	Bacterial actin homolog (cell-shape protein)
mRNA	Messenger RNA
MS	Mechanosensitive (e.g., MS channels)
MSCs	Mesenchymal stem/stromal cells
MscL	Mechanosensitive channel of large conductance
MscS	Mechanosensitive channel of small conductance
ms	Milliseconds
μg	Microgravity
µm	Micrometer
µN	Micronewton
nN	Nanonewton
NAPs	Nucleoid-associated proteins
NE	Nuclear envelope
Pa	Pascal
pN	Piconewton
PD(s)	Packing domain(s)
PRC2	Polycomb repressive complex 2
RNA	Ribonucleic acid
RNA-seq	RNA sequencing
s	Seconds
SMC	Structural Maintenance of Chromosomes (proteins/complexes)
TAD(s)	Topologically associating domain(s)
TAZ	Transcriptional coactivator with PDZ-binding motif (WWTR1)
TF	Transcription factor
TNNI3	Troponin I type 3 (cardiac) gene symbol
U937	U937 human myelomonocytic leukemia cell line
UFL	Private University of the Principality of Liechtenstein
Wsc1	Yeast cell-wall stress/mechanical sensor (cell-wall integrity pathway)
